# Aqueous Chemical Solution Deposition of Novel, Thick and Dense Lattice-Matched Single Buffer Layers Suitable for YBCO Coated Conductors: Preparation and Characterization

**DOI:** 10.3390/nano2030298

**Published:** 2012-09-10

**Authors:** Vyshnavi Narayanan, Sigelinde Van Steenberge, Petra Lommens, Isabel Van Driessche

**Affiliations:** Sol-Gel Centre for Research on Inorganic Powders and Thin Film Synthesis SCRiPTS, Department of Inorganic and Physical Chemistry, Ghent University, Krijgslaan 281-S3, Gent B-9000, Belgium; Email: sigelinde.vansteenberge@ugent.be (S.V.S.); petra.lommens@ugent.be (P.L.); isabel.vandriessche@ugent.be (I.V.D.)

**Keywords:** buffer layers, water-based precursors, chemical solution deposition, thin films, lattice-mismatch

## Abstract

In this work we present the preparation and characterization of cerium doped lanthanum zirconate (LCZO) films and non-stoichiometric lanthanum zirconate (LZO) buffer layers on metallic Ni-5% W substrates using chemical solution deposition (CSD), starting from aqueous precursor solutions. La_2_Zr_2_O_7_ films doped with varying percentages of Ce at constant La concentration (La_0.5_Ce*_x_*Zr_1−*x*_O*_y_*) were prepared as well as non-stoichiometric La_0.5+*x*_Zr_0.5−*x*_O*_y_* buffer layers with different percentages of La and Zr ratios. The variation in the composition of these thin films enables the creation of novel buffer layers with tailored lattice parameters. This leads to different lattice mismatches with the YBa_2_Cu_3_O_7−*x*_ (YBCO) superconducting layer on top and with the buffer layers or substrate underneath. This possibility of minimized lattice mismatch should allow the use of one single buffer layer instead of the current complicated buffer architectures such as Ni-(5% W)/LZO/LZO/CeO_2_. Here, single, crack-free LCZO and non-stoichiometric LZO layers with thicknesses of up to 140 nm could be obtained in one single CSD step. The crystallinity and microstructure of these layers were studied by XRD, and SEM and the effective buffer layer action was studied using XPS depth profiling.

## 1. Introduction

Chemical solution deposition (CSD) has been used as an important and cost-effective method for the deposition of YBa_2_Cu_3_O_7−*x*_ (YBCO) based second generation superconductors. *C*-axis oriented YBCO films for superconducting applications must be deposited on flexible, Rolling Assisted Biaxially Textured Substrates (RABiTS) of Ni-5% W in a “coated conductor architecture” [1]. The high temperature processing of YBCO layers (≥500 nm thickness), in an oxygen atmosphere can lead to the oxidation of the Ni-5% W substrate and penetration of Ni atoms from the substrate into the superconducting layer. In the event of such chemical interactions, the performance of the coated conductor will strongly decrease. Hence, buffer layers are absolutely necessary for coated conductor designs. Many buffer layers, including lanthanum zirconate (LZO) [2,3,4,5,6], cerium oxide (CeO_2_) [7,8,9,10] and other lattice-matched buffer layers [11,12,13,14,15,16,17,18,19,20] which are prepared basically using solution-based methods have been used. These buffer layers should not only stop diffusion but also transfer the epitaxial texture of Ni-5% W substrate to the superconducting layer. Of all the available buffer layers, LZO is used as a prominent buffer layer because of its cubic pyrochlore structure, which is stable enough to prevent the penetration of oxygen into the lattice. Its lattice parameter of 10.79Å provides a low lattice mismatch to both the orthorhombic YBCO (*a*-axis = 3.83 Å, *b*-axis = 3.89 Å) and the cubic Ni-5% W (*a*-axis = 3.54 Å) (~0.5% and 1.8% mismatch to YBCO, *a*- and *b*- axes, respectively and 7.6% to Ni-5% W substrate) [3].

However, an extra capping layer of CeO_2_ is preferred for better control of the bi-axial orientation of YBCO on the buffered metallic tapes [10], also due to the improved chemical compatibility as well as the improved nucleation of YBCO. Owing to the high cost of multiple buffer layer depositions of LZO and CeO_2_, researchers have focused on preparing a single buffer layer with almost 0% lattice mismatch with YBCO and it was reported that the in-plane orientation of YBCO can be improved with these lattice matched buffer layers [11,12,13,14,15,16,17,18].

In this research, La_2_Zr_2_O_7_ films doped with varying percentages of Ce at constant La concentration (La_0.50_Ce*_x_*Zr_1−*x*_O*_y_*) were prepared as well non-stoichiometric La_0.5+*x*_Zr_0.5−*x*_O*_y_* buffer layers with different percentages of La and Zr ratios. Ce^4+^ has a higher ionic radius (0.97 Å) than Zr^4+^ (0.86 Å) and its substitution in the Zr^4+^ lattice position of LZO results in a LCZO solid solution with a pyrochlore structure and increased lattice parameter with a very high thermal expansion coefficient [21]. Similarly, an increased percentage of La^3+^ in non-stoichiometric LZO (La_0.5+*x*_Zr_0.5−*x*_O*_y_*) leads to an increase of the lattice parameter values due to its higher ionic radius (1.16 Å) [12]. These variable compositions can lead to minimal lattice matches with the initially formed YBa_2_Cu_3_O_6+*x*_ which later turns to YBa_2_Cu_3_O_7−*x*_ under oxygen annealing conditions.

The influence of the precursor solution composition on the microstructure and crystallinity was studied using XRD and SEM. Their effective buffer layer action was studied using XPS depth profiling.

## 2. Materials and Methods

### 2.1. Preparation Method for Cerium Doped Lanthanum Zirconate (LCZO) Solutions

Acetic acid was added to zirconium n-propoxide (70% *w*/*w* in *n*-propanol, Sigma-Aldrich) in order to stabilize it (molar ratio Zr^4+^:HOAc = 1:4) [22]. This solution was lightly stirred. Cerium acetate (Ce(CH_3_COO)_3_·*x*H_2_O, Sigma-Aldrich, 99.9%), acetic acid and water ((Ce^3+^ + Zr^4+^):HOAc:H_2_O = 1:3:40) were added to this mixture. The partially dissolved mixture was heated to 60 °C. This was followed by the addition of lanthanum acetate (La(CH_3_COO)_3_·*x*H_2_O, Sigma-Aldrich, 99.9%), together with acetic acid and water ((La^3+^ + Zr^4+^ + Ce^3+^):H_2_O:HOAc = 1:7.5:100). The mixture was left to stir at 60 °C until all precipitates dissolved. In another beaker, ethylene diamine tetraacetic acid (EDTA, Sigma-Aldrich, 99.995%) solution was prepared. EDTA was used as the complexant. A specific molar ratio of the EDTA with respect to the total metal ions concentration (Ce^3+^ + La^3+^ + Zr^4+^):EDTA = 1:0.5) was dissolved in water at room temperature. The dissolution of EDTA in water was aided by increasing the pH of this mixture with ethylene diamine (EDA) (molar ratio of EDTA:EDA = 0.5:4). Later, both the metal ion solution and the EDTA solutions were mixed together and the pH was adjusted to 6 with addition of ammonia. A fixed amount of ethylene glycol (EG, molar ratio EDTA:EG = 1:4) was added to this solution under stirring and heated to 60 °C. The solution was evaporated until a concentration of 0.4 mol/L was obtained. According to the method described above, LCZO solutions with different percentages of zirconium and cerium (La_0.50_Ce*_x_*Zr_0.50−*x*_O*_y_* with “*x*” a variable amount) with a constant lanthanum percentage were prepared. The pHs of the solutions were found to be 6.0–6.2 and the viscosity 5.2–5.5 mPa s. Three different Ce^4+^ percentages were substituted in LZO systems leading to the following LCZO compositions: La_0.50_Ce_0.10_Zr_0.40_O*_y_*_, _La_0.50_Ce_0.25_Zr_0.25_O*_y_* and La_0.50_Ce_0.40_Zr_0.10_O*_y_*.

### 2.2. Preparation Method for Non-Stoichiometric Lanthanum Zirconate Solutions

The 0.4 mol/L non-stoichiometric LZO solutions were prepared by dissolving varying percentages of lanthanum acetate in a water-acetic acid mixture (molar ratio, La^3+^:HOAc:H_2_O = 1:16:150) at 80 °C until a clear solution was obtained. This was in accordance with the ratios satisfying the formula, La_0.5+*x*_Zr_0.5−*x*_O*_y_*. The water sensitivity of the zirconium alkoxide was reduced by stabilizing it with acetic acid (molar ratio, Zr^4+^:HOAc = 1:4) at room temperature [22]. This stabilized zirconium propoxide was diluted by adding water in a 1:20 molar ratio of Zr^4+^:H_2_O at 80 °C. A clear solution was obtained after stirring for 5 min at this temperature. Subsequently, the zirconium precursor solution was added to the lanthanum precursor solution at 80 °C. An EDTA solution (molar ratio, (La^3+^+Zr^4+^):EDTA = 1:0.5) was prepared by dissolving EDTA in water. The pH of this solution was increased by addition of ammonia until the dissolution of EDTA (pH between 7 and 8). This was then followed by the addition of the metal solution (La^3+^ + Zr^4+^) added to the EDTA solution at room temperature and stirred for 10 min. A viscous surfactant 2-amino-2-methyl-1-propanol (AMP, Sigma-Aldrich, 95%) with a high pH (>12) was added to the mixture (molar ratio, EDTA:AMP = 1:4) to increase the wettability and pH of this water-based precursor solution. The solution was heated to 60 °C. Finally, polyvinyl pyrrolidone (PVP) (molar ratio, (La^3+^ + Zr^4+^):PVP = 1:0.5) was added and the final solution evaporated until the desired concentration was achieved. The pH of the solution was found to be 4.6–4.8 and the viscosity 4.6–4.9 mPa s.

Two different La^3+^ and Zr^4+^ non-stoichiometric percentages were chosen that led to the following compositions: La_0.55_Zr_0.45_O*_y_* and La_0.60_Zr_0.40_O*_y_*.

### 2.3. Solution Stability, Influence of Complexant and Polymer

For both the non-stoichiometric LZO and the LCZO precursor solutions, the zirconium propoxide was stabilized by mixing it with acetic acid in order to handle it in non-vacuum and water-based conditions. Acetic acid partially replaces the propoxide (OPr) groups by acetate groups (OAc) forming a zirconium propoxide-acetate complex that reduces the chances of immediate hydrolysis and the condensation reaction of zirconium propoxide on the addition of water [22]. EDTA was used as a strong chelating agent for both precursors. It can form very strong complexes with lanthanum, cerium and zirconium ions [23], avoiding the precipitation of metal ions when high metal concentrations are reached during the transformation from solution to gel. For both precursors, additives are necessary to increase the viscosity and improve the wetting behavior over the metal substrate. EG was used in the LCZO solution preparation to increase the viscosity of the water-based solution. This allows the preparation of thicker layers from one single coating. A combination of AMP and PVP was used for the same reason in the non-stoichiometric LZO preparations. PVP is considered to promote structural relaxation during the heat-up stage of the annealing treatment thereby causing a reduction in stress during the formation of the layer and thus suppressing crack formation [24]. AMP acts as a surfactant but AMP and PVP cannot be used in the presence of Ce-precursor where they cause precipitation.

### 2.4. Cleaning of Substrates

Ni-5% W substrates of 2.5 cm by 1 cm and with a thickness of 80 μm (EVICO GmbH, Germany) were cleaned to remove contaminants and surface-treated to improve the wettability. Pre-treating the Ni-5% W substrates consists of chemical cleaning, etching and heat treatment under reduced gas conditions. Firstly, the substrate was degreased to remove the contaminants left over from the tape fabrication process [3]. This was done by dipping the metallic substrates for 5 min in each of the following solutions: isopropanol (Fiers, >99.7%), acetone (Fiers, >99.5%) and demineralized water. Each immersion lasts for a full 5 min to ensure the complete removal of all surface contaminants. After this degreasing and rinsing sequence, the substrates were exposed for 15 min to an etching mixture of hydrogen peroxide (H_2_O_2_, Carl Roth, >98%) and formic acid (HCOOH, Sigma-Aldrich, 35 wt %) in a 1:1 ratio at a temperature of 50–55 °C. The substrates were rinsed again with water and gently wiped with a fiber free tissue. Finally, the Ni-5% W RABiTS substrates were then placed into a quartz tube furnace under a reducing atmosphere of 5% H_2_ in Ar and a gas flow of 0.2 L/min. They were heated at a rate of 10 °C/min to 800 °C, dwelled at that temperature for 1 h and cooled to room temperature by turning off the furnace. The nickel and tungsten oxide species which might have formed at the surface during the etching step are reduced under these conditions and carried away by the gas flow.

### 2.5. Solution Deposition and Heat-Treatment

The cleaned Ni-5% W substrates were dip-coated in a clean room (class 10000; flow cupboard class 100) in order to prevent any contamination from dust particles. The dip-coat speed was varied between 20 and 60 mm/min. The as-deposited wet layers were transformed into a gel by placing them in a drying furnace at 60 °C for one hour. The amorphous layers in their gel state were subjected to a suitable heat treatment that resulted in a desired crystalline metal oxide phase in the following sequence: Firstly, the layers were heated from room temperature to 450 °C (ramp rate of 1 °C/min) and let to dwell for one hour. Secondly, a 3 °C/min heating ramp was applied from 450 to 900 °C (dwell time = 1 h). Finally, the films were heated to 1050 °C at a 10 °C/min ramp with a dwell time of 1 h. After the heat treatment, the furnace was switched off and the samples were left to cool inside the furnace. The entire heating process was carried out in an Ar-5% H_2_ atmosphere (gas flow rate: 0.1 L/min).

### 2.6. Characterization

The thickness of the crystallized layers was analyzed by spectroscopic ellipsometry (J.A. Woollam Co., USA) and by fitting the experimental curve to the model for LCZO films and non-stoichiometric LZO films on Ni-5% W substrates. The crystallinity of the film was analyzed using a standard X-ray diffractometer (Siemens D-5000). AXS Discoverer diffractometer was used to measure the phi scan of the buffer layers to quantify the in-plane grain misalignment. Analysis of the microstructure of the surface was performed with the help of a scanning electron microscope (FEG-SEM, FEI). The surface roughness of the layers was characterized using an atomic force microscope (AFM, Molecular Imaging Picoplus with Picoscan 2100 controller) in tapping mode. The buffer layer action was evaluated by means of monitoring the Ni penetration depth using X-ray Photoelectron Spectroscopy (S-Probe monochromatized XPS spectrometer, Surface Science Instruments (VG)), using an Al-K_α_ source (monochromatized Al-radiation: 1486.6 eV), at a base pressure of 2 × 10^−9^ mbar and an acceptance area of 250 × 1000 µm^2^, with a hemispherical analyzer at a pass energy of 157.7 eV. The measured surface was 250 μm by 1000 μm. The voltage of the Ar^+^-ion gun was maintained at 4 keV to sputter an area of 3 × 3 mm^2^. Experimental data were processed using the software package CasaXPS (Casa Software Ltd., UK) using Shirley background and Scoffield sensitivity factors.

## 3. Results and Discussion

### 3.1. Crystallinity and Orientation

**Figure 1:**
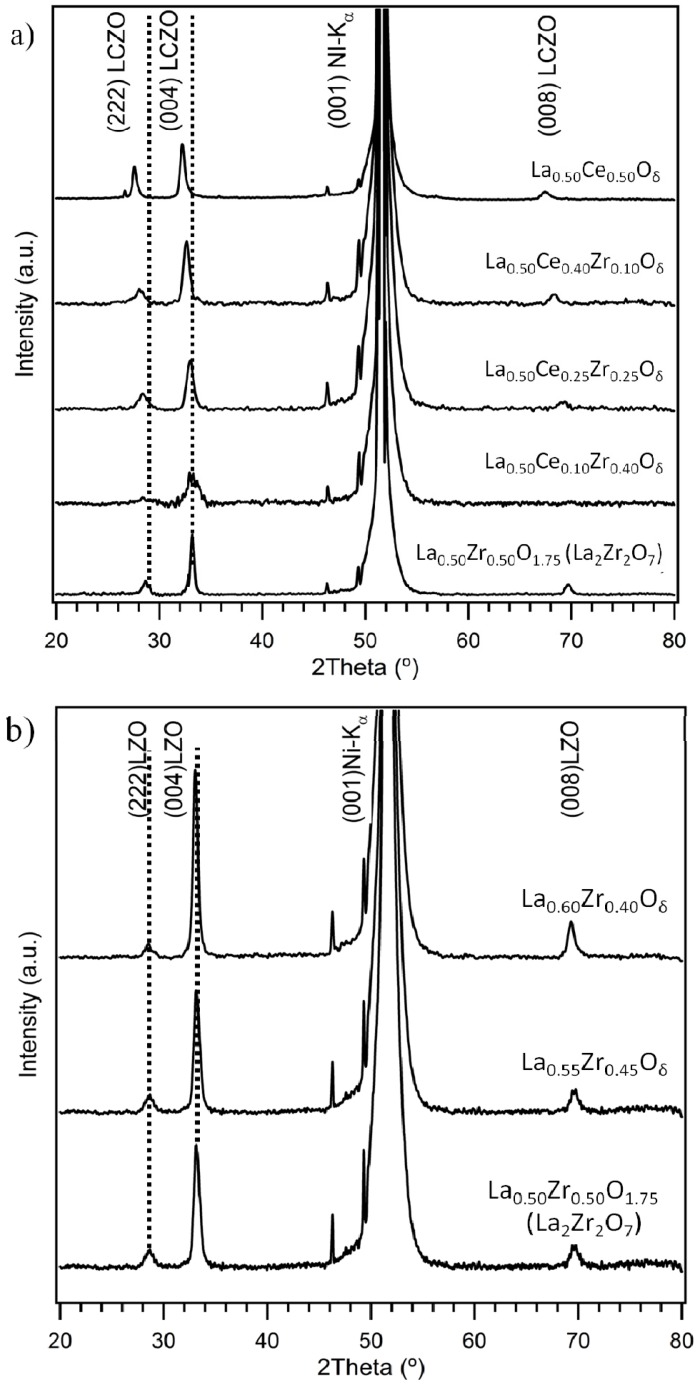
X-ray *θ*–2*θ* diffraction patterns obtained for (**a**) Cerium doped lanthanum zirconate (LCZO) buffer layers and (**b**) Non-stoichiometric lanthanum zirconate (LZO) buffer layers.

From ellipsometry, we find that at the maximum dip-coating speed of 40 mm/min, crack-free non-stoichiometric LZO layers of about 140 nm thick can be obtained. In the case of LCZO solutions, which have a slightly higher viscosity, the withdrawal speed is reduced to 20 mm/min, to achieve the same layer thickness. The epitaxial growth and crystallinity of these LCZO buffer layers and non-stoichiometric buffer layers of varying composition prepared from water-based precursor solutions were analyzed using XRD (Figure 1). For both systems, mainly reflections could be found, related to the epitaxially grown, *c*-axis oriented pyrochlore structure of stoichiometric LZO. In all samples, much weaker reflections related to the formation of a small fraction of the undesired (2 2 2) growth were also present. No additional reflections, indicative of the formation of secondary phases could be distinguished. For a stoichiometric LZO buffer layer with a pyrochlore structure, the (0 0 4) peak position normally is observed at 2*θ* = 33.3° and the less intense (0 0 8) peak position at 2*θ* = 69.8° [3]. In the case of the LCZO film, clearly the (0 0 4) and (0 0 8) reflections shift to lower angles as a function of Ce^4+^ doping percentage. This indicates that the larger Ce^4+^ ions are successfully doped into the LZO lattice, leading to an increase of the lattice parameter of the LCZO buffer layers, as can be expected from the larger ionic radius of Ce^4+^ (101 pm) compared to Zr^4+^ (86 pm). In the case of non-stoichiometric LZO, the peak shift is much more limited.

To determine the percentage of the preferential *c*-axis growth, the peak intensity ratio values were calculated as defined in Equation (1) [5].


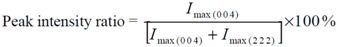
(1)

Accordingly, the percentage of the (0 0 *l*) oriented texture was calculated to be 91.00%, 80.25%, 76.50%, 78%, 66.35% for the LCZO films with increasing Ce content from *x* = 0.00, 0.10, 0.25, 0.40, 0.50, respectively. For the non-stoichiometric LZO films with increasing La content from *x* = 0.55, 0.60, the percentage of the (0 0 *l*) oriented texture was calculated to be 90.34%, 92.50%, respectively.

Although there is the desired *c*-axis oriented growth in the buffer layers, the unwanted (2 2 2) growth is also seen. This can be attributed to the fact that a probable bulk nucleation could have occurred and contributed to the undesired (2 2 2) oriented growth [5]. With bulk nucleation taking place within the film, the growth along the (0 0 *l*) plane will be affected.

**Figure 2:**
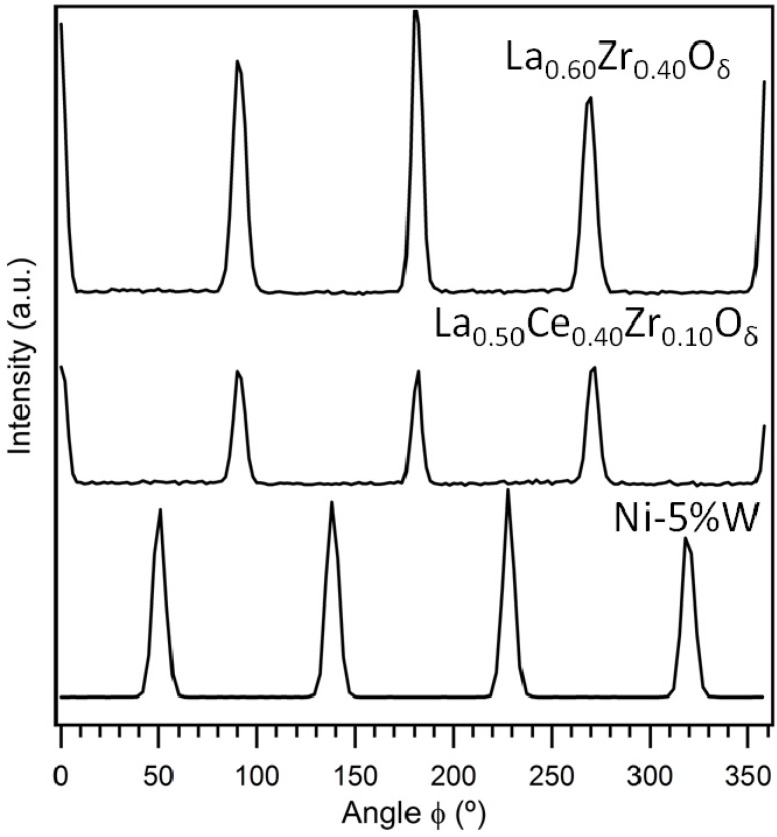
Phi-scan measurements of the (2 2 2) plane of the La_0.50_Ce_0.40_Zr_0.10_O*_y_* and La_0.60_Zr_0.40_O*_y_* buffer layers deposited on top of the Ni-5% W substrate in comparison to the (1 1 1) plane of the Ni-5% W substrate.

Figure 2 represents the phi-scans carried out to measure the in-plane grain misalignment of the (2 2 2) plane of the La_0.50_Ce_0.40_Zr_0.10_O*_y_* and La_0.60_Zr_0.40_O*_y_* buffer layers. From this figure it can be seen that the layers have clearly grown with a 45° rotation on top of the *c*-axis oriented Ni-5% W substrate indicating that the biaxial texture from the substrate was successfully transferred to the buffer layers. The average full width at half maximum (FWHM) of the reflections for the La_0.50_Ce_0.40_Zr_0.10_O*_y_* and La_0.60_Zr_0.40_O*_y_* buffer layers were found to be 7.62° and 6.75°, respectively.

#### 3.1.1. Lattice Mismatch Calculation for LCZO Thin Films on Ni-5% W

The YBCO superconducting thin film grows on top of LZO type buffer layers with a 45° rotation [16]. This minimizes the lattice mismatch between YBCO and the buffer layer. For the same reason, the buffer layer also rotates 45° with respect to the Ni-5% W substrate. Therefore, when discussing lattice matching, a so called pseudocubic lattice parameter is used, which is calculated using Pythagoras’s theorem from the actual cubic lattice parameters. For a stoichiometric LZO layer (*a* = 10.79 Å), e.g., the pseudocubic lattice parameter is calculated as in Equation (2),


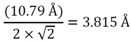
(2)

and the lattice mismatch with that of the underlying Ni-5% W (3.54 Å) substrate is calculated as follows in Equation (3):


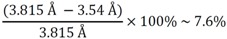
(3)

In Table 1, the lattice parameters as calculated from XRD for the range of LCZO samples is shown, together with the lattice mismatches with the Ni-5% W substrate and the initially formed YBCO_6 _*a*-axis.

**Table 1:** Lattice parameters of the La_0.50_Ce*_x_*Zr_0.5−*x*_O*_y_* thin films and their lattice mismatch with the Ni-5% W substrate and that of YBa_2_Cu_3_O_7−*x*_ (YBCO).

Sample	Lattice parameter *a* (Å)	Lattice mismatch with Ni-5% W (%) (3.54 Å)	Lattice mismatch with Y_1_Ba_2_Cu_3_O_6+*x*_ (%) (3.8578 Å)
La_0.50_Zr_0.50_O_1.75_	10.79	7.76	−1.11
La_0.50_Ce_0.10_Zr_0.40_O*_y_*	10.82	8.06	−0.84
La_0.50_Ce_0.25_Zr_0.25_O*_y_*	10.85	8.35	−0.58
La_0.50_Ce_0.40_Zr_0.10_O*_y_*	10.99	9.76	0.72

As can be seen, with increasing degree of substitution, the lattice mismatch with YBCO can be decreased and again increased. La_0.50_Ce_0.25_Zr_0.25_O*_y_* shows minimal lattice mismatch towards YBa_2_Cu_3_O_6+*x*_ (0.58%). Yet, it should be pointed out that introducing Ce also unavoidably leads to an increased lattice mismatch with the underlying Ni-5% W substrate, yet in XRD and in the phi-scan measurements, an acceptable range of epitaxial growth is observed. This shows that, an optimal doping of Ce needs to be chosen to compensate for the increased lattice mismatch with the substrate, as well as to obtain lower lattice mismatch with that of the YBa_2_Cu_3_O_6+*x*_ phase. Accordingly, La_0.50_Ce_0.25_Zr_0.25_O*_y_* can be chosen as a better composition. For the LCZO layers, the lattice parameter increased from 10.79 to 10.99 Å, upon replacing 80% of the Zr ions with Ce.

#### 3.1.2. Lattice Mismatch Calculation for Non-Stoichiometric LZO Thin Films on Ni-5% W

By increasing the La^3+^:Zr^4+^ ratio in the lanthanum zirconium oxide lattice (La_0.5+*x*_Zr_0.5−*x*_O*_y_*), the lattice parameter of the La_0.5+*x*_Zr_0.5−*x*_O*_y_* increases. Substitution until 60% of La^3+ ^is possible without disturbing crystal growth and inducing the formation of secondary phases. This is because fluorite also occurs, for more than 60% of La^3+ ^substitution, next to the pyrochlore phase [10]. Table 2 provides the lattice mismatch calculation for the prepared layers to that of Ni-5% W substrate and to different relevant YBCO lattice parameters.

**Table 2:** Lattice parameters of the La_0.5+*x*_Zr_0.5−*x*_O*_y_* thin films and their lattice mismatch with the Ni-5% W substrate and that of YBCO.

Sample	Lattice parameter *a* (Å)	Lattice mismatch with Ni-5% W (%) (3.54 Å)	Lattice mismatch with Y_1_Ba_2_Cu_3_O_6+*x*_ (%) (3.8578 Å)
La_0.50_Zr_0.50_O_1.75_	10.79	7.76	−1.11
La_0.55_Zr_0.45_O*_y_*	10.83	8.20	−1.06
La_0.60_Zr_0.40_O*_y_*	10.89	8.39	−0.99

La_0.60_Zr_0.40_O*_y_*, e.g., shows lower lattice mismatch to that of the Y_1_Ba_2_Cu_3_O_6+*x *_*a*-axis which might lead to high quality YBCO growth on top of these buffer layers. For non-stoichiometric LZO, a shift from 10.79 to 10.89 Å is calculated when moving from stoichiometric LZO to La_0.60_Zr_0.40_O*_y_*.

### 3.2. Microstructural Analysis

**Figure 3:**
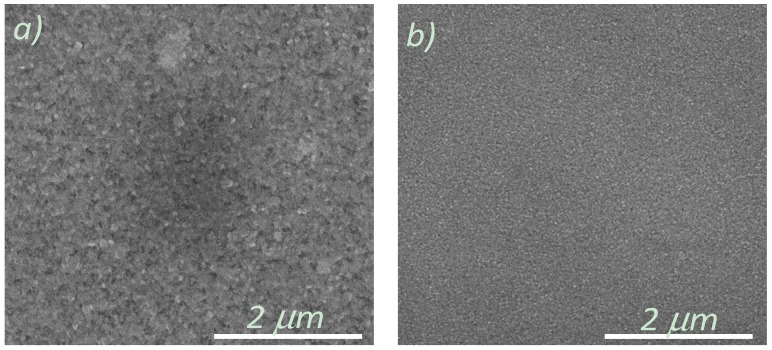
Scanning electron microscope (SEM) image of (**a**) La_0.50_Ce_0.40_Zr_0.10_O*_y_* on Ni-5% W substrate; (**b**) La_0.60_Zr_0.40_O*_y_* on Ni-5% W substrate.

A dense surface morphology is essential for a buffer layer in order to prevent the diffusion of nickel atoms into the YBCO layers as well as to prevent the oxidation of the Ni-5% W substrate that takes place during physical deposition of YBCO or during heat-treatment of the CSD YBCO layers. Therefore, SEM analyses were performed to understand the morphology of the top surface of the layers made from all four systems. The scanning electron microscopy studies carried-out on these layers reveal a dense and crack-free surface morphology as shown in Figure 3a,b. This kind of dense surface morphology is required to prevent any diffusion of Ni into the YBCO layer.

**Figure 4:**
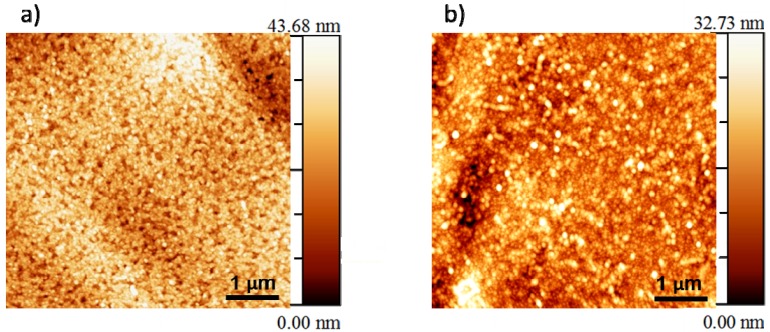
Atomic force microscope (AFM) image of (**a**) La_0.50_Ce_0.40_Zr_0.10_O*_y_*, (**b**) La_0.60_Zr_0.40_O*_y_* on Ni-5% W substrate.

It is also necessary to create buffer layers with a surface having low roughness, in order to be able to obtain high quality YBCO growth on top [9]. Accordingly, AFM analysis was performed on the layers of La_0.50_Ce_0.40_Zr_0.10_O*_y_* and La_0.60_Zr_0.40_O*_y_* layers on Ni-5% W substrate to quantify the surface roughness, as shown in Figure 4.

AFM image for a La_0.50_Ce_0.40_Zr_0.10_O*_y_* on Ni-5% W substrate has a RMS roughness of 6.868 nm. On the other hand, for the La_0.60_Zr_0.40_O*_y_* the RMS roughness was found to be 4.862 nm [25]. The surface roughness values are comparable to those of earlier reported values of surface roughness for water-based LZO layers [2,5].

### 3.3. Buffer Layer Action: XPS Study

**Figure 5:**
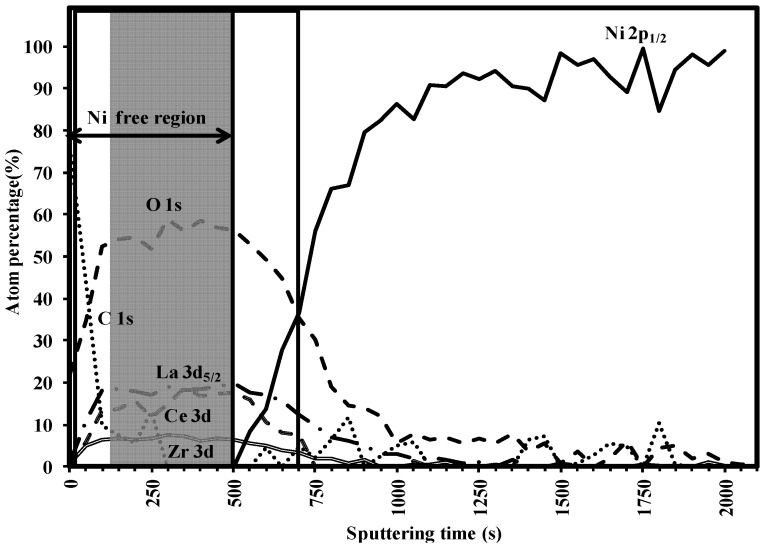
X-ray photoelectron spectroscopy (XPS) depth profile spectrum for La_0.50_Ce_0.40_Zr_0.10_O*_y_* layer.

The capacity of the buffer layers to withstand nickel penetration can be evaluated by XPS depth profiling [26]. Figure 5 shows the result of 40 consecutive sputter cycles, each consisting of an elemental analysis of C, La, Ce, Zr, Ni, O after 50 s of Ar^+^ ion bombardment for an as-deposited La_0.50_Ce_0.40_Zr_0.10_O*_y_* layer. The layer under study has a thickness of 120 nm as obtained from ellipsometry.

To estimate the effective buffer layer thickness that prevented Ni penetration it was assumed that, the estimated buffer layer thickness can be simulated to the concentration of the percentage of La dropping below half of its original value (as seen in the depth profile spectrum Figure 5). In this La_0.50_Ce_0.40_Zr_0.10_O*_y_* layer, the concentration of the La drops below half of its original after 700 s of sputtering. Thus it can be roughly calculated that, 90 nm is sputtered in 550 s or we have a sputtering rate of 1.78 Å/s. The Ni penetration starts from 500 s of sputtering. Thus, the nickel-free zone on the top of the sample is approximately 90 nm. The thickness of the penetration zone is estimated to be ~30 nm. Accordingly, it can be confirmed that the La_0.50_Ce_0.40_Zr_0.10_O*_y_* acts as a good barrier layer against Ni penetration.

**Figure 6:**
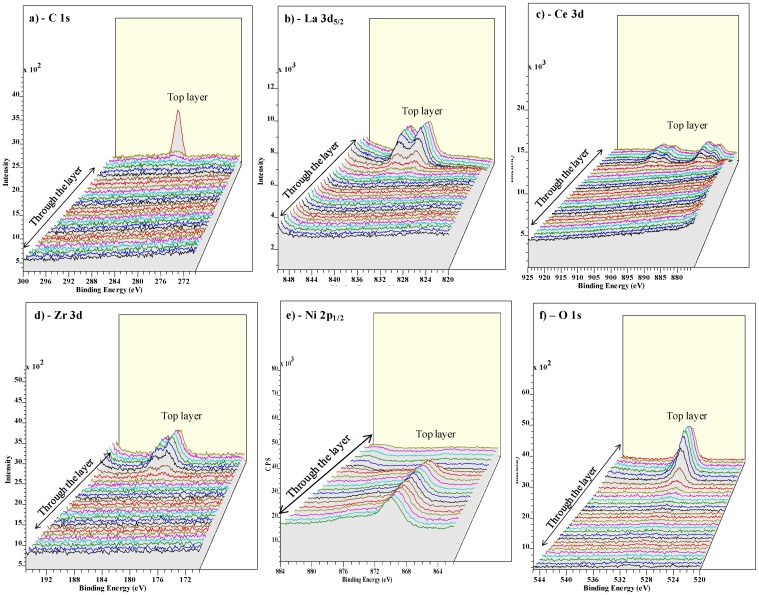
XPS overview spectrum of the various elements through the La_0.50_Ce_0.40_Zr_0.10_O*_y_* layer.

From Figure 6a, it can be seen that, the C is seen more prominently in the top layer. The La peaks shift through the layer, which can be attributed to the varying oxygen and hence the state of La which is shifting through the layer (Figure 6b). The similar argument holds for Ce and Zr peaks in Figure 6c,d. The Ni peak is clearly seen to rise with decreasing La, Ce and Zr concentrations with the increasing sputtering time (Figure 6e). Figure 6f shows that oxygen is predominantly seen until the buffer layers are seen and decreases constantly as the Ni peak rises.

To summarize it can be stated that, Ce doping in LZO provides tailor-made compositions with lower lattice mismatch to that of the initially formed Y_1_Ba_2_Cu_3_O_6+*x*_ on top of it. However with increased Ce doping, the lattice mismatch of the buffer layer with that of the underlying Ni-5% W substrate increases. Nevertheless, there is an optimal Ce doping composition (La_0.50_Ce_0.25_Zr_0.25_O*_y_*) which provides better lattice mismatch to that of Y_1_Ba_2_Cu_3_O_6+*x*_ and with an agreeable epitaxial growth that is transferred from the substrate. The epitaxial growth can also be improved with some changes in the heat-treatment conditions as there is a significant amount of carbon still present in the top 10 nm of the layer (Figure 5). Moreover, focus on improved smoothness of the top surface of these kinds of buffer layers will also be taken care of in future research.

## 4. Conclusions

This work presents the preparation and characterization of the cerium doped LZO (La_0.50_Ce_1−*x*_Zr_0.50−*x*_O*_y_*) and the non-stoichiometric LZO lanthanum zirconium oxide (La_0.5+*x*_Zr_0.5−*x*_O*_y_*) buffer layers as well as the shift in lattice parameter that was observed for these doped layers with varying stoichiometries. The lattice parameters of these layers were tunable according to the lattice parameters of YBCO to provide less lattice mismatch. The SEM analysis revealed a dense morphology which is necessary for a good buffer layer. The XPS study showed that the buffer layer was effective in preventing the diffusion of Ni atoms.
